# Resveratrol and Calcium Signaling: Molecular Mechanisms and Clinical Relevance

**DOI:** 10.3390/molecules19067327

**Published:** 2014-06-05

**Authors:** Audrey E. McCalley, Simon Kaja, Andrew J. Payne, Peter Koulen

**Affiliations:** 1 Vision Research Center, Department of Ophthalmology, School of Medicine, University of Missouri—Kansas City, 2411 Holmes St., Kansas City, MO 64108, USA; E-Mails: aemqp4@mail.umkc.edu (A.E.M.); kajas@umkc.edu (S.K.); paynea@umkc.edu (A.J.P.); 2 Department of Basic Medical Science, School of Medicine, University of Missouri—Kansas City, 2411 Holmes St., Kansas City, MO 64108, USA

**Keywords:** resveratrol, calcium, signaling, inflammation, pain, cancer, Alzheimer’s disease, Huntington’s disease, Parkinson’s disease, and amyotrophic lateral sclerosis, cardiovascular disease, diabetes mellitus, autoimmune

## Abstract

Resveratrol is a naturally occurring compound contributing to cellular defense mechanisms in plants. Its use as a nutritional component and/or supplement in a number of diseases, disorders, and syndromes such as chronic diseases of the central nervous system, cancer, inflammatory diseases, diabetes, and cardiovascular diseases has prompted great interest in the underlying molecular mechanisms of action. The present review focuses on resveratrol, specifically its isomer *trans*-resveratrol, and its effects on intracellular calcium signaling mechanisms. As resveratrol’s mechanisms of action are likely pleiotropic, its effects and interactions with key signaling proteins controlling cellular calcium homeostasis are reviewed and discussed. The clinical relevance of resveratrol’s actions on excitable cells, transformed or cancer cells, immune cells and retinal pigment epithelial cells are contrasted with a review of the molecular mechanisms affecting calcium signaling proteins on the plasma membrane, cytoplasm, endoplasmic reticulum, and mitochondria. The present review emphasizes the correlation between molecular mechanisms of action that have recently been identified for resveratrol and their clinical implications.

## 1. Introduction

### 1.1. Effects and Mechanisms of Action of Resveratrol as the Basis for Its Therapeutic Potential in Various Diseases

Resveratrol is a stilbenoid commonly found in the roots of Japanese Knotweed and the skin of red grapes. Plants produce phytoalexin as a response to harmful stimuli such as UV radiation, infection, or other pathogenic threats [1,2]. Increased curiosity about resveratrol and its potential health benefits began with a phenomenon termed the “French Paradox” [3] that became a fascination of health professionals and researchers. The “French Paradox” describes the extremely low incidence rate of cardiovascular disease in France compared with other European countries despite a high fat diet with little to no exercise [4]. Epidemiological studies indicated that polyphenols present in red wine could be responsible for the cardioprotective effects experienced by the French [4,5,6]. Resveratrol then initially became a subject of study for potential cardiovascular benefits but additional benefits are becoming apparent in recent studies [7]. Some of the proposed mechanisms include antioxidant, anti-carcinogenic, anti-inflammatory, anti-aging [8], and anti-nociceptive functions indicating the potential of resveratrol as therapeutic agent for preventing and ameliorating a wide range of pathologies [9,10,11,12,13,14], including neurodegenerative diseases such as Alzheimer’s disease (AD), Huntington’s disease (HD), Parkinson’s disease (PD), and amyotrophic lateral sclerosis (ALS) [8,15,16]. Cardiovascular diseases that may benefit from resveratrol treatment include hypertension, ventricular arrhythmia, myocardial infarction (MI) induced ventricular tachycardia, ventricular fibrillation, arteriosclerosis, arteriolosclerosis, and restenosis particularly after angioplasty [7,13,17,18,19,20]. In addition, cerebral ischemia, diabetes mellitus, cancer, autoimmune diseases, and other inflammatory-related issues have been discussed as potentially benefitting from resveratrol intervention [13,15,21,22]. Resveratrol also provides protection from UV radiation explaining its potential use in the prevention of age-related macular degeneration (AMD) [23,24]. Potential mechanisms underlying resveratrol's actions are its ability to control protein activity via interaction with transmembrane and intracellular enzymes [25].

### 1.2. Key Signaling Proteins Controlling Cellular Calcium Homeostasis

Resveratrol is a potent regulator of genomic and non-genomic processes including regulation of membrane potential, DNA transcription, enzyme activity, secretion, apoptosis, mitochondrial activity, and intracellular ion homeostasis, including the modulation of the intracellular calcium concentration [3,7,25]. 

Resveratrol can act as a ligand for trans-membrane proteins [23] including voltage-gated calcium channels (VGCC) and plasma membrane calcium ATPase (PMCA) [26,27]. Intracellularly, calcium release activated channels (CRAC), sarco-/endoplasmic reticulum calcium ATPase (SERCA), and intracellular calcium channels (ICC) contribute to calcium ion homeostasis [28,29,30,31,32]. Resveratrol potentially increases endoplasmic reticulum (ER) calcium concentrations through modulation of SERCA but may also contribute to the decrease or stabilization of the release of calcium from intracellular stores by modulating ICCs [31,33,34,35,36]. Most importantly, there is significant evidence to conclude that resveratrol contributes to overall calcium homeostasis during states of cellular dysfunction [37]. While resveratrol does not exhibit cellular toxicity, mild systemic yet reversible gastrointestinal disturbances were detected when resveratrol was used for extended periods of time in high concentrations [38,39]. 

## 2. Medical Relevance of Modulation of Cellular Calcium Signaling Mechanisms by Resveratrol

### 2.1. Modulation of Cellular Calcium Signaling Mechanisms by Resveratrol in Excitable Cells

Resveratrol potently modulates the intracellular calcium concentration in excitable cells through a variety of mechanisms that control calcium influx, store filling, release from intracellular stores, and downstream activation of calcium sensitive molecules. In myocytes, resveratrol increases the refractory period and decreases the threshold for membrane depolarization [17]. This results in restored calcium homeostasis evident by a reduction of delayed after depolarization (DAD, occurs with cytoplasmic calcium influx) and triggered activity (TA, ensues via irregular calcium release) in myocytes [37,40,41]. Resveratrol, therefore, can ameliorate cardiac arrhythmia and prevent premature atrial contraction (PAC) [17]. 

Similar membrane hyperpolarization in response to resveratrol also occurs in smooth muscle cells (SMC) leading to vasodilation, making resveratrol a candidate for the treatment of a wide variety of disorders including hypertension, ventricular arrhythmia, myocardial infarction (MI) induced ventricular tachycardia, and ventricular fibrillation [18,41,42]. Furthermore, it has been suggested that the ability of resveratrol to induce endothelium-dependent hyperpolarization of SMCs may compensate for pathologic absence or dysfunction of endothelium nitric oxide synthase (eNOS) and cyclooxygenase-1 (COX-1) [43]. Endothelial cells line the walls of the lymphatic and cardiovascular vessels and are critical modulators of contractility and vessel dimensions. Vasorelaxation is the most extensively studied effect of resveratrol on vascular endothelia. Most studies focus on two interlinked mechanisms of action, the direct effect on the intracellular calcium concentration and the downstream indirect effects on cellular potassium regulation [44]. A resveratrol dependent increase in nitric oxide biosynthesis was shown to result from calcium mobilization rather than direct agonism of eNOS by the drug [45]. The major plasma membrane targets of resveratrol include the L-type VGCC, which is directly inhibited by resveratrol, and the large conductance calcium-activated potassium (BK) channel, that resveratrol indirectly effects [19,37]. Under physiological conditions, calcium influx via L-type VGCCs stimulates BK channels that facilitate cytosolic potassium ion efflux, which results in membrane hyperpolarization. Resveratrol directly attenuates calcium influx, indirectly decreasing potassium efflux, and leading to a state of vasorelaxation in an endothelium-dependent manner [20,46,47]. Due to these effects, resveratrol may be beneficial in preventing or mitigating the physiological effects of hypertension, arteriosclerosis, arteriolosclerosis, and restenosis (particularly after angioplasty). Interestingly, diabetes mellitus is an important, clinically relevant risk factor for all four conditions [48]. 

**Figure 1:**
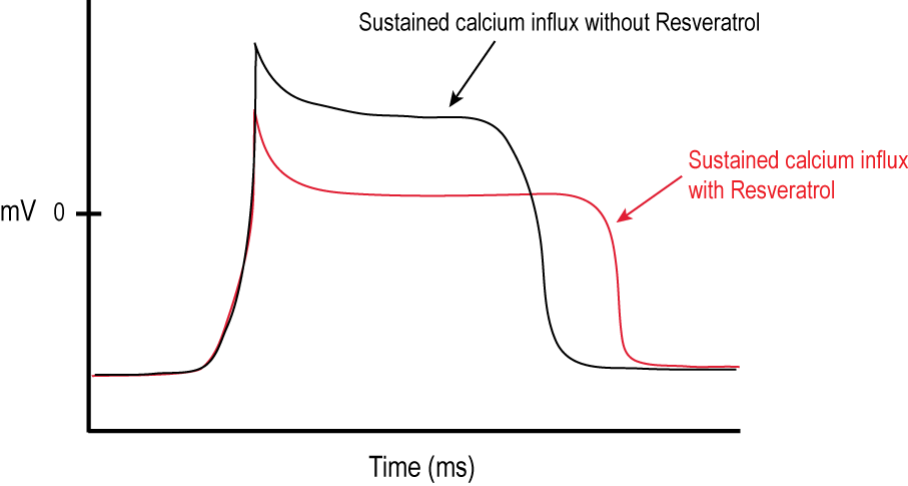
**Resveratrol’s mechanism of action in excitable cells**. Resveratrol lowers the threshold and increases the duration of calcium influx in excitable cells [16,17].

In neuronal cells, resveratrol reduces the action potential threshold and both delays and prolongs calcium entry (Figure 1) [16]. This effect causes rapid sodium-induced action potentials that increase neuronal conduction. This mechanism of action has the potential to regulate and increase neural impulses which may provide a basis for the prevention of conductance disorders or to retard neurodegenerative processes. Resveratrol has also been shown to protect against cerebral ischemic damage via indirect BK channel activation, thereby preventing neuronal hyperexcitability and the ensuing cell death [15,16]. While resveratrol can prevent neuronal death and provide protection against oxygen-glucose deprivation [49,50], the potential of resveratrol for therapeutic intervention in axonopathy or other conduction block disorders remains to be elucidated [8,51]. The pleiotropic effects of resveratrol with its respective molecular mechanisms, and the pharmacological modulation of those mechanisms, affect separate components of membrane depolarization and repolarization, resulting in a distinct modulation of spatio-temporal properties of electrical signaling by resveratrol in excitable cells.

### 2.2. Modulation of Cellular Calcium Signaling Mechanisms by Resveratrol in Cancer and Immune Cells

Resveratrol shows great promise as a therapeutic agent for a variety of cancers [52]. Given its effects on calcium homeostasis, resveratrol may be useful as a latent remedy for the primary stages of some malignant cancers [53]. Calcium signaling pathways are necessary for the motility and invasive characteristics of malignant tumors, as it controls cell restructuring and dedifferentiation [54]. Another potential mechanism of action for resveratrol targets the excessive energy demand of tumor cells, lowers calcium thresholds, and prolongs intracellular calcium surges, all of which ultimately lead to cell death as a result of mitochondrial dysfunction [55]. Previously, resveratrol has been shown to suppress CD4^+^CD25^+^ regulatory T cells (T_reg_ cells) in a preclinical cancer model [56]. T_reg_ are critical mediators of self-tolerance and inhibit the proliferation of CD4^+^CD25^−^ conventional T-cells (T_con_) through a recently identified mechanism involving the suppression of intracellular calcium release [57]. Further studies are needed to identify how resveratrol modulates intracellular calcium homeostasis during the early phases of tumor growth.

**Figure 2:**
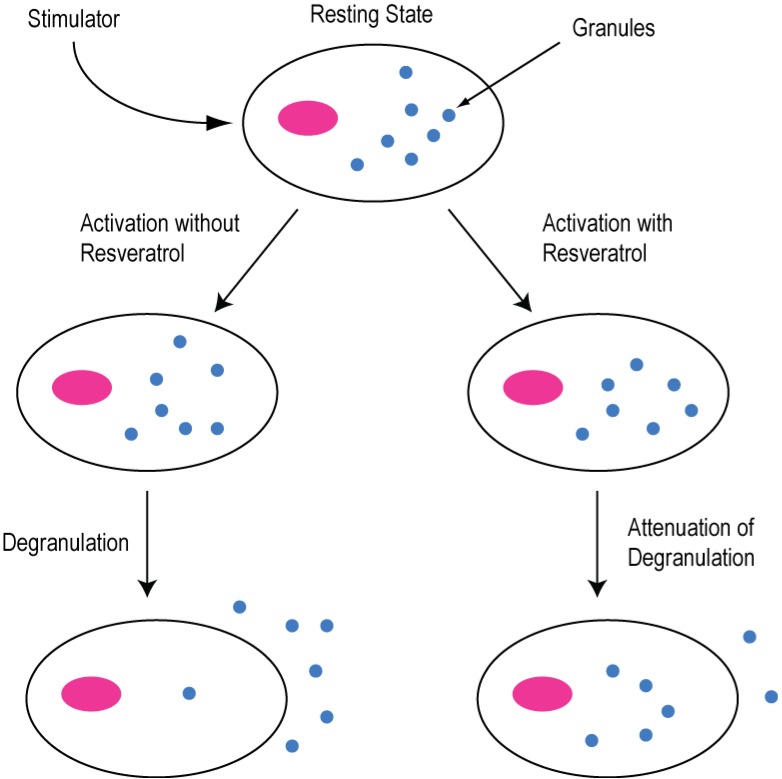
**Resveratrol attenuates immune responses related to degranulation**. Resveratrol decreases calcium influx, inhibiting granule release in mast cells [14,58].

Resveratrol is a powerful modulator of the immune system response via both pro- and anti-inflammatory pathways [58,59]. Resveratrol has the ability to activate immune cells at low doses while exerting an inhibitory effect at high doses [21,52]. The mechanism of action of resveratrol in immune cells likely involves a combination of proteasome inhibition [60] and modification of the cytosolic calcium concentration through blocking calcium entry and increasing calcium storage [31]. It has been suggested that resveratrol can reversibly modify disturbed system responses such as mast cell granulation [56]. Resveratrol decreases mast cell hypersensitivity by reducing calcium influx thereby reducing granulation (Figure 2) [12]. Resveratrol has also been shown to decrease inflammatory responses in autoimmune disorders, such as multiple sclerosis, through potentiation of active T-cell apoptosis [56,61]. It appears that resveratrol exerts effect on intracellular calcium by modulating sequestration and release of intracellular stores but further studies are needed to elucidate the exact mechanism of action in lymphocytes and other cell types of the immune system.

### 2.3. Modulation of Cellular Calcium Signaling Mechanisms by Resveratrol in Human Retinal Pigment Epithelial Cells

**Figure 3:**
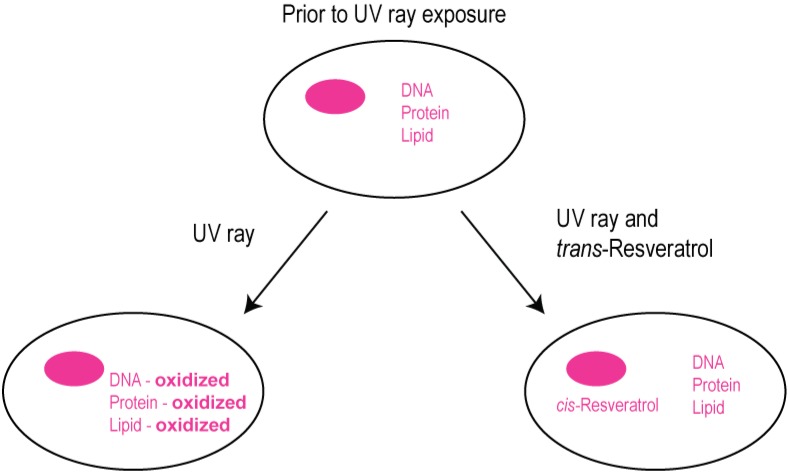
**Resveratrol protects RPE cells from UV damage**. *Trans*-resveratrol absorbs UV light and is converted to *cis*-resveratrol thereby preventing RPE cells from the deleterious effects of UV damage [23,62,63,64].

*Trans*-resveratrol is susceptible to photoisomerization to *cis*-resveratrol when exposed to ultraviolet irradiation [62]. When present in retinal pigment epithelial (RPE) cells, or any cell subject to UV ray exposure, resveratrol absorbs damaging UV light, thus reducing exposure of other structures to damaging wavelengths and ultimately enhancing the protective function of RPE cells (Figure 3) [23]. One key mechanism in RPE cells is phagocytosis, an important renewal mechanism that is susceptible to UV damage [23]. In experimental studies, resveratrol preserved RPE cell phagocytosis when applied prior to insult but had no protective effect when used as a post-insult treatment [23]. It has been proposed that this protective effect is mediated with BK channels [23]. Chronic resveratrol supplementation, rather than acute administration, would therefore be required for pharmaceutical intervention studies. 

Another protection mechanism includes the anti-oxidative effect of resveratrol [63,64]. Pretreatment of RPE cells with resveratrol ameliorated the oxidative stress related damage caused by hydrogen peroxide by preventing oxidation related RPE phagocytic impairment, which may be linked to BK channel activity [23]. Furthermore, the clinical course of age-related macular degeneration (AMD), and potentially other vascular diseases of the retina, may be slowed by resveratrol when given as a nutritional additive [24,65].

## 3. Therapeutic Potential of Resveratrol via the Modulation of Cellular Calcium Signaling

### 3.1. Intracellular Calcium Channels

Two main types of intracellular calcium channels (ICC), inositol 1,4,5-trisphosphate receptors (IP_3_Rs) and ryanodine receptors (RyRs), are vital for the release of calcium from the endoplasmic and sarcoplasmic reticulum (ER & SR). Release of intracellular calcium through IP3Rs increases the intracellular calcium concentration which in turn activates RyRs to amplify the calcium signal — a process termed calcium induced calcium release (CICR). ICCs are involved in fundamental cellular processes such as cell proliferation, signaling, excitability, gene expression, and apoptosis [66]. Thus, ICCs are considered a potential target for neuroprotective and cytoprotective strategies for a number of pathologies [28,66,67] although, to date, no studies have been targeted at identifying the specific effects of resveratrol on ICCs (Table 1). Understanding the potential effects of resveratrol on ICCs could identify novel treatment strategies for neurodegenerative diseases such as Alzheimer’s disease (AD), Huntington’s disease (HD), amyotrophic lateral sclerosis (ALS), and other neurodegenerative diseases. 

### 3.2. Store-Operated Calcium Channel

Calcium-release activated channels (CRACs) are store-operated calcium channels located at the plasma membrane that are activated by low intracellular calcium store concentrations, resulting in a sustained increase of the cytosolic calcium concentration. Immune cells rely on this mechanism and CRACs are potential drug targets for anti-inflammatory diseases [68]. Resveratrol has not been shown to exert a direct effect on CRAC channels [69] (Table 1) although it has been suggested that resveratrol acts indirectly on store-operated calcium entry (SOCE). However, the potency of resveratrol stimulation on CRAC is far lower than that of other known neuroprotective and neuromodulatory compounds including nonsteroidal estrogens [33].

### 3.3. Voltage-Gated Calcium Channels

Plasma membrane voltage-gated calcium channels (VGCCs) are classified into high or low voltage-activated channels. VGCCs are activated by a depolarization of the membrane potential and allow extracellular calcium flux into the cytosol, thereby translating an electric signal into a secondary chemical signal. The class of high VGCCs include Ca_V_1 (L-type) and Ca_V_2 (P/Q-, N-, and R-type) channels. The low VGCCs includes Ca_V_3 (T-type) channels. Ca_V_1, Ca_V_2, and Ca_V_3 have slow, intermediate, and fast inactivation responses, respectively. In excitatory cells, resveratrol decreases cellular excitability by attenuating calcium influx through VGCCs [70]. Diseases related to VGCC dysregulation include neurodegenerative disorders such as AD, PD, cerebral ischemia, cardiovascular diseases, chronic and acute inflammatory diseases, many cancers, and even mood disorders [28,45,71]. The primary mechanism of action of how VGCC dysregulation leads to these disorders is hyperexcitability resulting in a chronic increase in the cytosolic calcium concentration. Therefore, VGCCs, especially the slow inactivating Cav1 channels, have become a focus of neuroprotective strategies [28,72]. Resveratrol has been shown to dose-dependently inhibit both L-type and T-type calcium channels and to increase the time period for reactivation [29]. More specifically, resveratrol inhibits L-type currents during phase 2 of an action potential, leading to vasorelaxation [26]. Also, it has been suggested that resveratrol-induced vasorelaxation is partially due to a stimulation of NOS and the ensuing chronic increase of the nitric oxide concentration [30,41]. 

### 3.4. Calcium-Activated Potassium Channels

Calcium-activated potassium (K_Ca_) channels include three major types of channels- large (BK), intermediate (IK), and small conductance (SK) channels. K_Ca_ channels help regulate the action potential activity of excitable cells via the modulation of membrane hyperpolarization. These channels play a critical role in restricting calcium influx [73]. Resveratrol does not appear to act directly on K_Ca_ channels but rather exerts its effects through other types of calcium channels. This higher level regulation of calcium homeostasis in turn indirectly regulates potassium flux [19,47,74]. While there is no general consensus, most studies suggest that the modulation of K_Ca_ channels by resveratrol’s inhibitory effects illustrates an indirect partial reduction of potassium efflux due to moderation of calcium influx [9,75]. 

**Table 1:** Resveratrol’s interactions with cellular proteins and its effects on components of the cellular calcium signaling machinery.

Protein	Modulatory Action	Therapeutic Application	References
Intracellular calciumchannels	TBD	Potential for excitatory neuronal, cardiac, inflammatory and autoimmune diseases	---
Calcium-release activated channels	No direct effects	No direct disease amelioration	[60]
Store-operated calcium entry	Activation of store-operated calcium entry	Diseases of prolonged calcium influx such as immune and inflammatory diseases	[33]
Voltage-gated calcium channels	Dose-dependent inhibition of L- and T-type channels	Prevention of uncontrolled excitability	[26,27]
Calcium-activated potassium channels	Indirect inhibition, likely via modulation of voltage-gated calcium channels	Modulation of action potentials (particularly in cardiac and neurological disorders)	[19,47,74]
SERCA	Indirect up-regulation via SIRT1 activation	SIRT1 down regulation disorders, cancer	[26,27]
PMCA	Indirect PMCA degradation via calpain activation	Cancer	[27]

### 3.5. SERCA and PMCA

Calcium ATPases are calcium transport proteins that return the cellular calcium concentrations back to the transmembrane electrochemical gradient state prior to renewed depolarization. The two key proteins that mediate this mechanism are the plasma membrane calcium ATPase (PMCA), which pumps calcium out of the cell when present in high concentrations, and the SR calcium ATPase (SERCA), which replenishes the SR calcium store from the cytoplasm. To our knowledge there is no study investigating potential effects of resveratrol on PMCA. However, it appears that resveratrol upregulates SERCA via SIRT1 activation, further replenishing calcium stores and reducing intracellular calcium influx [62]. Resveratrol may prove useful to attenuate diseases related to SIRT1 downregulation [26]. Additionally, resveratrol facilitates apoptosis in cancerous cells by induction of a calpain-dependent PMCA degradation mechanism. This strategy may be further elucidated through experiments targeted at identifying resveratrol’s mechanism of action in tumor cells [27]. 

### 3.6. Mitochondrial Calcium Signaling

Mitochondria are temporary and rapidly releasing calcium stores, capable of generating calcium spikes or waves to trigger second messenger systems. Mitochondria are critical initiators of apoptotic pathways via calcium signaling. Resveratrol has been shown to induce mitochondrial apoptosis by alteration of the mitochondrial membrane potential and eliciting a mitochondrial permeability transition (MPT) pore by lowering the calcium threshold necessary for MPT opening [27,55,76]. This mechanism is currently intensively investigated as a potential for anti-cancer drug therapy [77,78]. For instance, it has been proposed that resveratrol can induce this apoptosis in cancerous cells due to differences in mitochondrial function and that it can be injected directly into a tumor mass to initiate tumor regression prior to resection [79].

## 4. Conclusions

Resveratrol is a potent modulator of many cellular calcium signaling pathways (Table 1). The number of different targets for modulation by resveratrol (Table 1) confound the interpretation of *in vivo* analyses and result in cell type- and organism-specific responses. While L-type and T-type VGCCs have been identified as direct targets of resveratrol modulation, further studies are required to identify the effects on intracellular calcium channels and other neurotransmitter receptor systems. Yet there is significant evidence to suggest that the beneficial effects of resveratrol cannot be attributed only to its immunomodulatory function but also to a resveratrol dependent lowering of membrane hyperexcitability and cellular calcium toxicity. Thus resveratrol bears great promise for a number of multifactorial pathologies including neurodegeneration, autoimmune and cardiovascular disease, and cancer. Further, resveratrol as should be considered both an immunomodulator and a modulator of intracellular calcium signaling. 
